# Interbody cage use on successful spinal correction in pedicle subtraction osteotomy for adult spinal deformity surgery: a systematic review and meta-analysis of comparative studies

**DOI:** 10.1007/s43390-025-01218-5

**Published:** 2025-10-30

**Authors:** Omkar S. Anaspure, Aryan S. Anaspure, Anthony N. Baumann, Tensae Assefa, Nnaemeka Okorie, David Casper, Amrit S. Khalsa

**Affiliations:** 1 Perelman School of Medicine, University of Pennsylvania, 3400 Civic Center Blvd, Philadelphia, PA 19140 USA; 2 School of Arts and Sciences, Rutgers University, New Brunswick, NJ USA; 3 Department of Orthopaedic Surgery, Cleveland Clinic Akron General, Akron, OH USA; 4 Department of Orthopaedic Surgery, University of Pennsylvania, Philadelphia, PA USA

**Keywords:** Adult spine deformity, Schwab grade 3 osteotomy, Schwab grade 4 osteotomy, Interbody cage, Pedicle subtraction osteotomy, Bone–disc–bone osteotomy

## Abstract

**Introduction:**

Adult spinal deformity (ASD) often necessitates high-grade osteotomies to achieve adequate correction and durable arthrodesis, yet guidance on adjunctive interbody cage use remains fragmented. This systematic review aims to aggregate and critically appraise clinical and radiographic outcomes of interbody cage use in Schwab grade 3 and 4 osteotomies to provide evidence-based guidance for complex ASD correction.

**Methods:**

We conducted a PROSPERO-registered systematic review and meta-analysis (CRD420251068532) of comparative studies to evaluate perioperative characteristics of ASD patients undergoing corrective high-grade osteotomies. We queried PubMed, EMBASE, and CINAHL databases through June 7th, 2025. Inclusion criteria were studies that examined ASD patients who underwent high-grade osteotomy and reported postoperative metrics such as blood loss, final degree of correction, and complication rates. A random-effects binary and continuous model for meta-analysis was performed using risk ratios.

**Results:**

Four comparative studies (*n* = 367; 50.41% male; mean age 57.75 ± 11.1 years) were included. Baseline mean preoperative sagittal vertical axis (SVA), Visual Analog Pain scores (VAS), and Oswestry Disability Index scores (ODI) for the total cohort were 19.40 cm ± 10.90 cm, 57.26 ± 25.13, and 61.91 ± 17.74, respectively. There was no significant difference in operative time regardless of cage use (mean time Cage: 461.76 ± 158.65 min; No Cage: 452.47 ± 169.04 min, mean difference (MD): 22.01; CI [− 13.92, 57.94] *p* = 0.23). There was no significant difference in intraoperative blood loss regardless of cage use (mean Cage: 1,576.29 ml ± 483.58 ml; No Cage: 1,513.64 ml ± 844.41 ml; MD: 49.53; CI [− 127.65, 226.72], *p* = 0.584). Osteotomies with cage use had a significantly lower postoperative SVA compared to cageless osteotomies (mean Cage SVA: 5.20 ± 2.55 cm; No Cage: 5.81 ± 2.50 cm; *p* = 0.03). There was no significant difference in ODI regardless of cage use (mean ODI Cage: 29.04 ± 5.84; No Cage: 35.42 ± 5.46 cm; *p* = 0.116). When comparing outcomes between patients with or without cages, no differences were seen in instances of rod fractures (6.94% vs 4.64%), proximal junctional kyphosis (15.61% vs 14.9%), or proximal junctional failure (4.05% vs 4.12%) respectively (*p* > 0.05).

**Conclusion:**

ASD patients who underwent high-grade osteotomies with an interbody cage had lower postoperative SVA compared to patients who underwent cageless osteotomies. No differences were seen in operative time, blood loss, complication rates, or patient-reported outcomes between groups. Cage use may benefit patients at higher risk for nonunion or mechanical failure. Further prospective studies are needed to better define the role of cages in ASD correction.

## Introduction

Adult spinal deformity (ASD) is characterized by sagittal or coronal plane deformity in skeletally mature adults leading to significant pain, disability, and diminished quality of life. While nonoperative management is appropriate for mild or nonprogressive cases, surgery is indicated for severe or progressive deformity with objectives of restoration of global alignment, decompression of neurologic elements, and attainment of a solid fusion construct [1–3]. In deformities unamenable to less invasive techniques, spinal osteotomies are employed for achieving adequate correction. Selection between three-column osteotomies and multilevel low-grade approaches depends on rigidity, desired correction magnitude, and morbidity considerations [2–6].

High-grade osteotomies such as Schwab grade 3 pedicle subtraction osteotomies (PSO) and grade 4 bone–disc–bone osteotomies (BDBO) are imperative for correcting severe, rigid ASD with fixed sagittal or multiplanar imbalance that cannot be addressed by lower-grade techniques [3, 6, 7]. PSO achieves approximately 30–40° of angular correction via a wedge resection at a single level, whereas BDBO extends the resection into the adjacent disc and endplates to provide even greater correction when the deformity apex lies at the disc space [6–9]. Achieving robust arthrodesis across osteotomies is necessary to maintain the realigned construct and prevent recurrence or hardware failure. This is generally accomplished through meticulous decortication of osteotomy surfaces, application of autograft or allograft bone, and rigid posterior fixation to support fusion.

Devices such as interbody cages may further augment construct stability and can contribute to higher fusion rates and are generally used in procedures such as lumbar interbody fusions. Currently, no universal guidelines exist regarding the use of interbody cage use in Schwab grade 3 and 4 osteotomies for ASD. The recent study by Mullins et al. (2025) indicates that anterior column support via cages at the osteotomy site can significantly enhance fusion rates (79.2% vs. 55.1%) and segmental lordosis correction without increasing perioperative complications. Concerns persist, however, regarding increased operative time, cost, technical complexity, and challenges in patients with prior fusions or poor bone quality [5, 10].

Present guidance on interbody cage use during high‐grade osteotomies in ASD remains greatly.

fragmented, with heterogeneous data on patient‐reported outcomes, fusion success, and complication profiles. In response to this lack of consensus, there has been a growing body of comparative and single‐cohort studies exploring the efficacy of cages in promoting arthrodesis, enhancing segmental alignment, and sustaining construct durability within high-grade osteotomies in this population. No systematic review to date has comprehensively evaluated these clinical and radiographic outcomes across Schwab grade 3 and 4 osteotomies. By formally aggregating and appraising this gap in the literature, our review seeks to provide spine surgeons with rigorous, evidence-based guidance on the utility of interbody cages as an adjunct to three-column osteotomies, thereby informing surgical strategy and optimizing patient outcomes in complex ASD correction.

## Methods

### Study registration and guideline adherence

This study is a systematic review of comparative studies examining the use of interbody cages versus no cages in Schwab Grade 3–4 osteotomies for patients with ASD. This study was pre-registered on the International Prospective Register of Systematic Reviews (PROSPERO) prior to the article sorting process (CRD420251068532). Additionally, this study was conducted under the guidance of the Preferred Reporting Items for Systematic Reviews and Meta-Analysis (PRISMA) guidelines.

### Study creation with search strategy

This systematic review searched PubMed, EMBASE, and CINAHL from database inception until June 7th, 2025, using the following search algorithm: (“Pedicle Subtraction Osteotomy” OR grade “4 osteotomy” OR three column osteotomy OR “bone-disc-bone osteotomy” OR “Pedicle Subtraction Osteotomy with a Cage” OR “Schwab grade IV osteotomy” OR “lumbar spinal osteotomies and spinopelvic fixation”).

### Inclusion and exclusion criteria

Inclusion criteria included full-text English articles with patients who underwent spinal osteotomy for surgical correction of ASD pathologies using either a Schwab Grade III or IV classification procedure. Studies were only included if at least one group received placement of an interbody cage. All patients must be 18 years of age or older. Exclusion criteria included articles without full-text, not in English, case reports, unclear osteotomy approach, or those involving non-ASD patients.

### Study group definitions

**Fig. 1:**
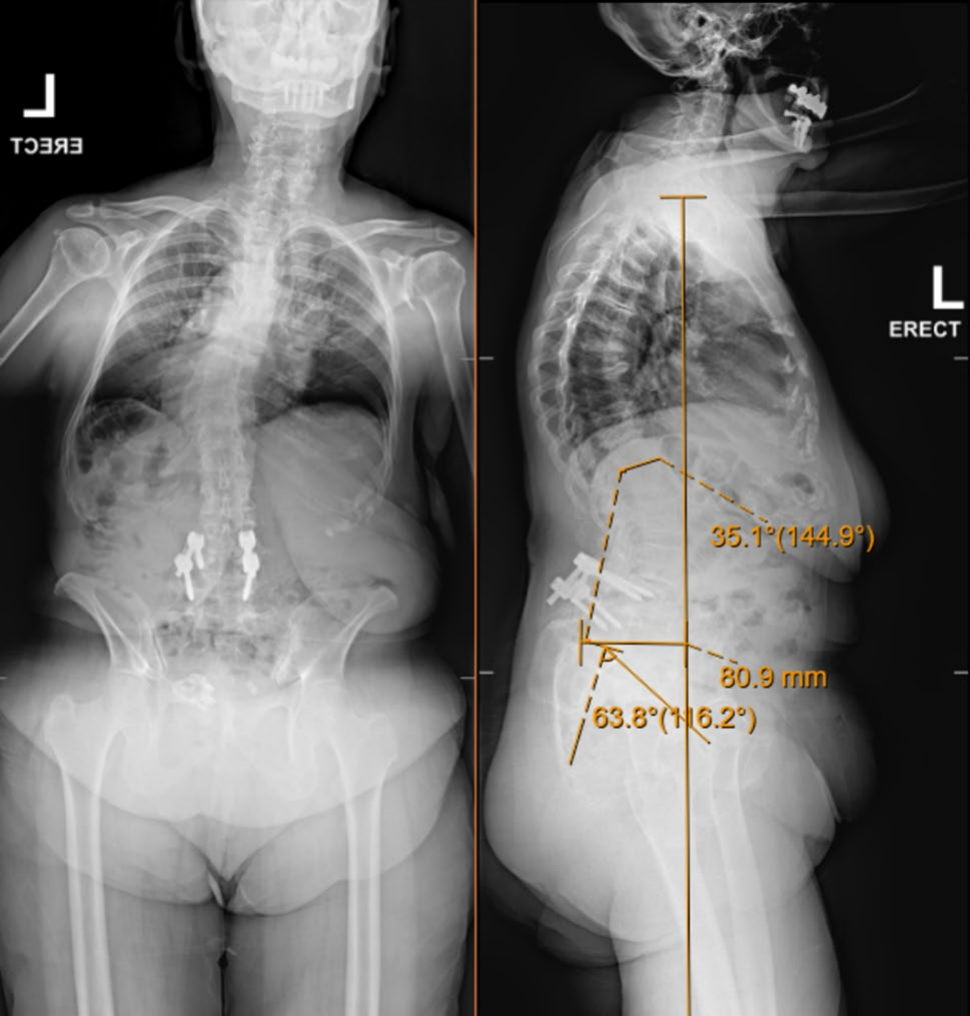
74-year-old patient with a history of L4-5 posterolateral fusion presenting with left-sided low back pain, difficulty with upright posture, and progressive lower extremity paresthesias and weakness secondary to spinal stenosis. Preoperative X-rays (PA and lateral) of the thoracolumbar spine show sagittal imbalance with SVA 8.9 cm and Pelvic incidence—Lumbar Lordosis mismatch of ~ 28 degrees. Retained implants in segmental kyphosis at L4-L5 and previous L5-S1 fusion

**Fig. 2:**
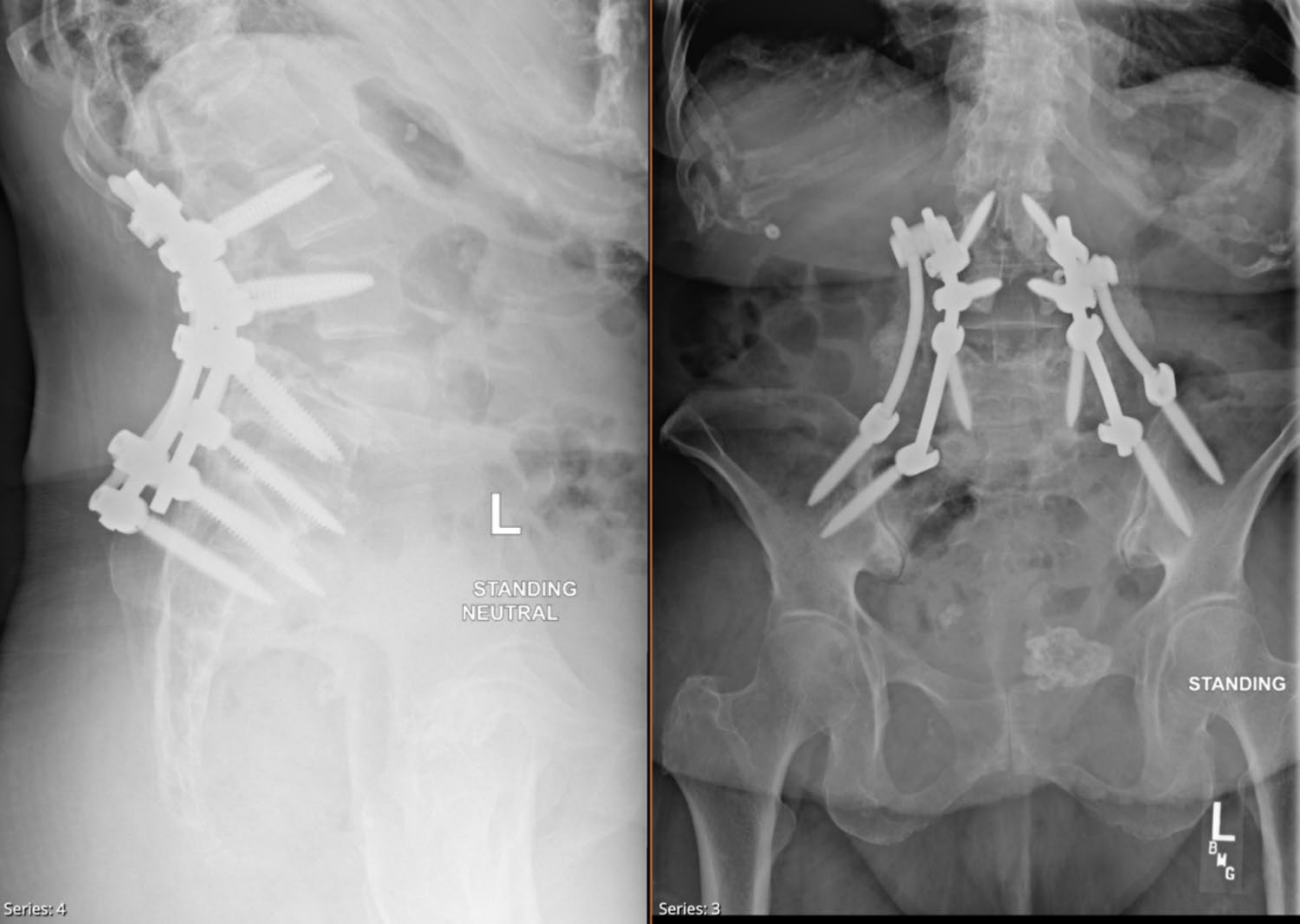
Lumbar spine radiographs (AP and Lateral) at final follow-up after L2–pelvis posterior spinal fusion with revision L3–L4 laminectomy and L4 pedicle subtraction osteotomy

This study defines an osteotomy as “high grade” if it meets SRS-Schwab Grade III or SRS-Schwab Grade IV classification for spinal osteotomies as seen in the literature [11]. This grading is used to characterize osteotomies by the number of levels removed and location within the vertebral column. For the purpose of this study, we considered Schwab Grade III osteotomies if surgeons used a PSO, which involves a posterior-based three-column osteotomy at a single vertebral level. This procedure generally includes resection of the posterior elements (lamina, pedicles, and facets), decortication of the vertebral body, and removal of the posterior vertebral wall, allowing for closure of the osteotomy through hinging on the anterior cortex [8, 12, 13]. An example of this is provided in Figs. 1 and 2 as courtesy of A.S.K to show pre- and postoperative imagine following PSO. We characterized procedures as Schwab Grade IV if the osteotomy followed the BDBO approach, which extends the resection to include the posterior elements, the entire vertebral body, and the adjacent superior or inferior intervertebral disc [8, 12–14].

### Article selection process

The article screening process was completed by a single author with the final decision to include being made by the second author. After the initial database search, all retrieved articles were uploaded into Rayyan to facilitate the sorting process [15]. First, duplicate articles were removed and then articles were sorted by title and abstract. Next, articles were sorted by full-text. Upon the final inclusion of any articles, a full reference search of the included articles was completed.

### Data extraction process

Data extraction was completed by a single author with final edits made by the second author. Then, once a final data set was obtained, another author independently verified all of the data for accuracy with any discrepancies resolved by the second author after communication. Data extracted included first author, year of publication, number of patients, sex, operative time, blood loss, patient reported outcomes (PROs), postoperative complications, and radiographic parameters of adequate correction.

### Article quality grading

All observational studies included in this systematic review were classified as either “comparative” or “non-comparative” to appropriately grade their quality using the Methodological Index for Non-Randomized Studies (MINORS) scale [16]. Comparative studies were graded out of 24 points and non-comparative studies were graded out of 16 points. For comparative studies, there are 8 items on the scale, and for non-comparative studies, there are 12 items on the scale with each item being rated from 0 to 2 points. All articles were considered to be “high-quality”, “moderate-quality”, or “low-quality” based on their scoring and comparative/non-comparative nature. For comparative studies, high-quality articles scored 24 points, moderate-quality articles scored 15–23 points, and low-quality articles scored less than 15 points [17]. For non-comparative studies, high-quality articles scored 16 points, moderate-quality articles scored 10–15 points, and low-quality articles scored less than 10 points [17].

### Statistical synthesis

This study utilized the Statistical Package for the Social Sciences (SPSS) version 29.0 (Armonk, NY: IBM Corp) for statistical analysis. Descriptive statistics such as frequency weighted means (FWM) and percentages were used where no statistical significance could be calculated. A random-effects model was chosen for the meta-analysis to account for the inherent variability between studies (e.g., differences in study populations, methodologies, or sample sizes), ensuring that the results reflect both within-study and between-study variation. This approach was used to provide more generalized conclusions, given the heterogeneity expected among observational studies. The random-effects binary outcomes meta-analysis was conducted using relative risk (RR) and 95% confidence intervals (CIs) to compare binary outcomes across studies. Instances of zero complications were adjusted to 0.5 in binary outcomes, as noted in other literature [18, 19]. Meta-analysis for continuous outcomes was performed by utilizing the means for specific outcomes with the respective standard deviation values. To further improve comprehensiveness, studies reporting means with missing standard deviations (SD) were supplemented with the SD from the next closest study in terms of means, sample sizes, and/or study type as seen elsewhere in the literature [20]. Meta-analysis was only done if more than two studies reported the respective outcomes. A forest plot was created to illustrate the relationships between variables and was shown only for the intraoperative time and intraoperative blood loss outcomes.

## Results

### Initial search results and article grading

**Fig. 3:**
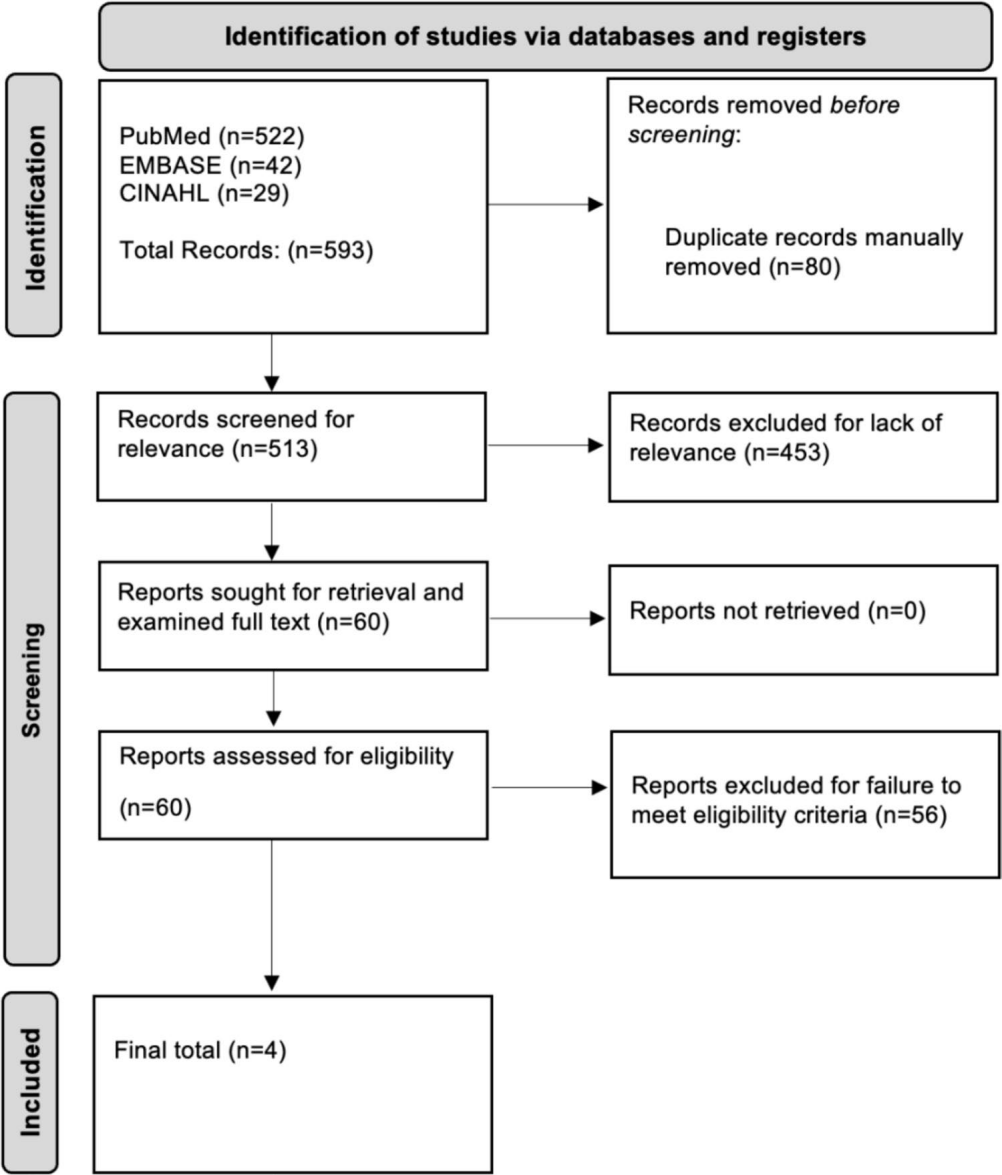
The Preferred Reporting Items for Systematic Reviews and Meta-Analyses diagram

The database search resulted in 593 articles; after manual de-duplication, 513 articles remained. After title and abstract screening, 60 articles were included in full-text analysis. Ultimately, four total articles met inclusion criteria and were included in the data extraction process [21–24] (Fig. 3).

### Article quality results

Of the 4 included articles, all were comparative and retrospective in nature. Mean MINORS score for comparative studies was 19 (out of 24.0 points). Ultimately, all included articles were "moderate quality," with no "high quality" or “low quality” articles included in this study (Table 1).

**Table 1:** The methodological index for non-randomized studies (MINORS) grading for all included articles in this systematic review

First author (year)	Study type	Total MINORS Score	Clearly stated aim	Inclusion of consecutive patients	Prospective collection of data	End points appropriate to study aim	Unbiased assessment of study end point	What Follow-up period appropriate to study aim	Less than 5% lost to follow up	Prospective calculation of the study size	Adequate control group	Contemporary groups	Baseline equivalence of groups	Adequate statistical analysis
Li et al. (2022)	Comparative	19	2	2	0	2	1	2	2	0	2	2	2	2
Hu et al. (2018)	Comparative	19	2	2	0	2	1	2	2	0	2	2	2	2
Rostami et al. (2025)	Comparative	19	2	2	0	2	1	2	2	0	2	2	2	2
Mullins et al. (2025)	Comparative	19	2	2	0	2	1	2	2	0	2	2	2	2

### Study baseline characteristics

Patients (*n* = 367) had a FWM age of 57.75 ± 11.1 years and comprised 185 males and 182 females (Table 2). At baseline, the mean preoperative sagittal vertical axis (SVA), Visual Analog Pain scores (VAS), and Oswestry Disability Index scores (ODI) for the total cohort were 19.40 cm ± 10.90 cm, 57.26 ± 25.13, and 61.91 ± 17.74, respectively (Table 3). Key findings and takeaways from each study can be found in Table 2. There was no statistically significant difference in these baseline characteristics between the cage and no cage group across all four individual studies.

**Table 2:** Patient baseline characteristics from all four articles and summarized key findings

Author (year)	Groups	Specific procedure subtype	No. of patients	Mean age (years)	Sex	Summarized key findings
Li et al. (2022)	Interbody cage	Grade IV Osteotomy	11	61.7 ± 6.1	Male (*n* = 3), Female (*n* = 8)	There was no significant difference in Cobb angle, SVA, or ODI between the 2 groups (*p* > 0.05). There were no complications, such as spinal cord injury, internal fixation loosening, or fracture, or deaths in the study
	No cage		13	61.7 ± 6.1	Male (*n* = 5), Female (*n* = 8)	
Hu et al. (2018)	Interbody cage	PSO	43	43.1	Male (*n* = 36), Female (*n* = 7)	Cage group achieved the same realignment of the spine as the cageless group. PSO with a cage significantly avoided sagittal translation during the osteotomy procedure and might represent a new, safe, and feasible choice for treating patients with AS kyphosis
	No cage		46	46.5	Male (*n* = 41), Female (*n* = 5)	
Rostami et al. (2025)	Interbody cage	Grade III or IV osteotomies	71	58.6 ± 12.9	Male (*n* = 25), Female (*n* = 46)	Improved postoperative sagittal vertical axis and pelvic tilt were noted in the cage group (*p* < 0.01). Both VAS and ODI scores were significantly improved at 6 and 12 months in the cage group (*p* < 0.01). No significant differences in estimated blood loss or operation time were observed. The use of cages significantly reduces rod fracture rates, improves sagittal alignment, and clinical outcomes in ASD surgery
	No cage		86	61 ± 7	Male (*n* = 38), Female (*n* = 48)	
Mullin et al. (2025)	Interbody cage	Grade III or IV osteotomies	48	63.5 ± 8.0	Male (*n* = 20) Female (*n* = 28)	Cage use was associated with a larger mean change in segmental lordosis at the osteotomy site (32.9 ± 9.6 vs. 28.7 ± 9.5, *p* = 0.038). Fusion rates were significantly higher in the cage group (79.2% vs. 55.1%, *p* = 0.0012). Overall, cage use was associated with improved fusion rates and greater segmental lordosis without increasing complication rates. Incorporating cages may provide enhanced alignment and fusion outcomes in complex ASD surgeries
	No cage		49	66.7 ± 8.3	Male (*n* = 17), Female (*n* = 32)

**Table 3:** Radiographic outcomes, subjective patient-reported outcomes, and final correction evaluation following high-grade osteotomy by cage use for four included studies

Author (year)	Groups	SVA Preop (cm)	SVA Postop (cm)	VAS Preop	VAS Postop	ODI Preop	ODI Postop	Adequate final correction
Li et al. (2022)	Interbody cage	5.8 ± 2.2	1.0 ± 0.3	6.6 ± 1.6	1.1 ± 0.5	70.8 ± 17.0	25.9 ± 8.7	100% (*n* = 11/11)
	No cage	6.0 ± 3.4	1.6 ± 0.8	5.8 ± 1.5	1.2 ± 0.7	63.2 ± 15.5	27.1 ± 10.0	100% (*n* = 13/13)
Hu et al. (2018)	Interbody cage	25.1 ± 15.2	6.8 ± 7.6	–	–	–	–	100% (*n* = 43/43)
	No cage	26.4 ± 14.5	7.5 ± 8.2	–	–	–	–	100% (*n* = 46/46)
Rostami et al. (2025)	Interbody cage	20.5 ± 6.81	4.41 ± 1.84	62.6 ± 16.7	34.3 ± 8.8	69.1 ± 14	25.1 ± 10.7	–
	No cage	21.98 ± 7.11	5.93 ± 1.33	67.1 ± 15.8	42.8 ± 10.6	66.8 ± 17.1	35.6 ± 11.1	–
Mullin et al. (2025)	Interbody cage	14.34 ± 7.45	5.9 ± 6.56	–	–	51.8 ± 15.6	35.6 ± 16.2	–
	No cage	13.29 ± 5.95	5.14 ± 6.86	–	–	50.5 ± 16.0	37.3 ± 20.2	–

### Total intraoperative time by cage use

**Fig. 4:**
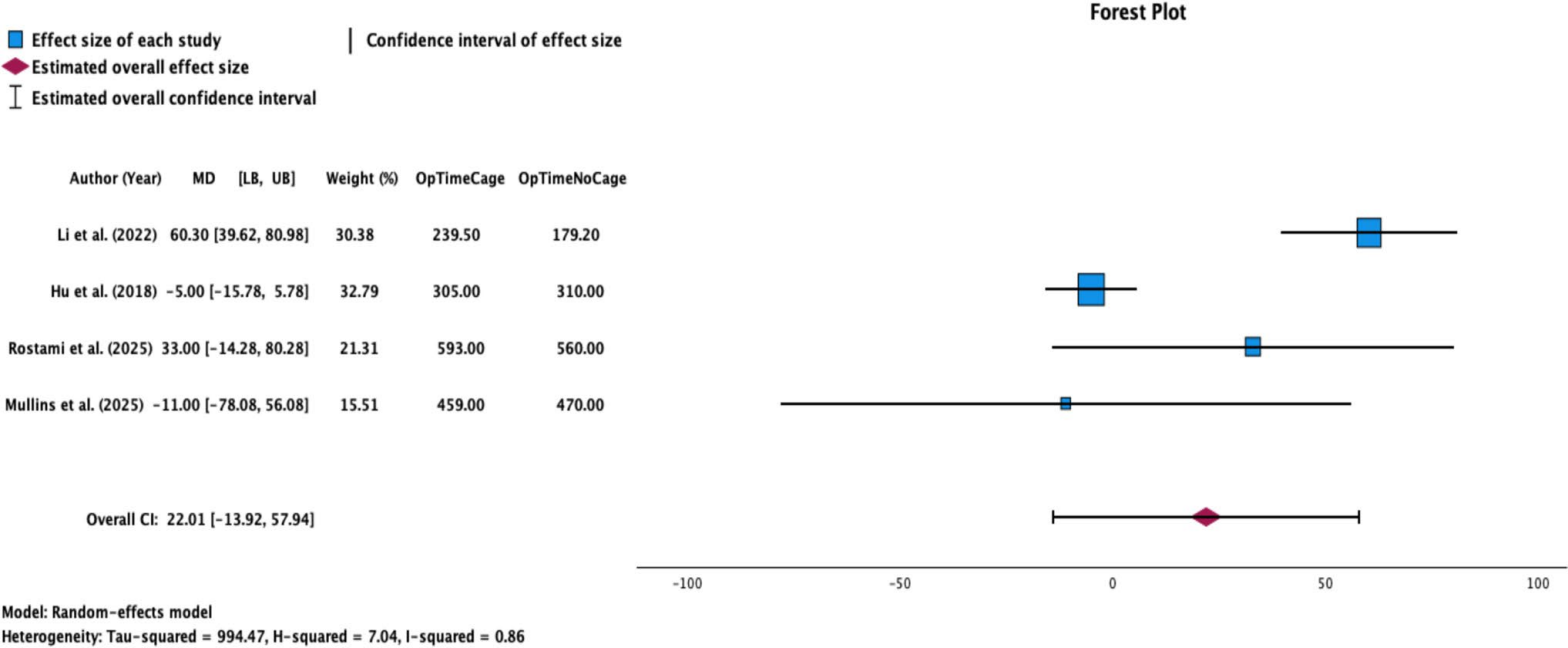
Random-effects model forest plot demonstrating the relationship between operative time and cage use for four studies. Effect measures and CIs were calculated for both individual study outcomes and reported as overall pooled outcomes. *LB* lower bound, *UB* upper bound. Heterogeneity: Tau-squared = 994.47, H-squared = 7.04, I-squared = 0.86. The axis is shown using a linear scale

The FWM operative time for osteotomies with cage was 461.76 ± 158.65 min and the FWM operative time for cageless osteotomies was 452.47 ± 169.04 min [21–24]. When examining operative time for high-grade osteotomies stratified by cage use via a random-effects meta-analysis, we found that there was no difference regardless of cage use across the four studies (*p* = 0.230; Overall effect size: 22.01; 95% CI [− 13.92, 57.94]; Fig. 4**, **Table 4).

**Table 4:** Mean operative time and blood loss by cage use

Author (year)	Groups	Mean operative time (min)	Blood loss (mL)
Li et al. (2022)	Interbody cage	239.5 ± 29.0	1,560.9 ± 378.6
	No cage	179.2 ± 22.7	1,242.3 ± 339.0
Hu et al. (2018)	Interbody cage	305	700
	No cage	310	780
Rostami et al. (2025)	Interbody cage	593 ± 151	1276 ± 391
	No cage	560 ± 150	1258 ± 364
Mullin et al. (2025)	Interbody cage	459 ± 174	2809 ± 2152
	No cage	470 ± 163	2723 ± 2022

### Total estimated blood loss by cage use

**Fig. 5:**
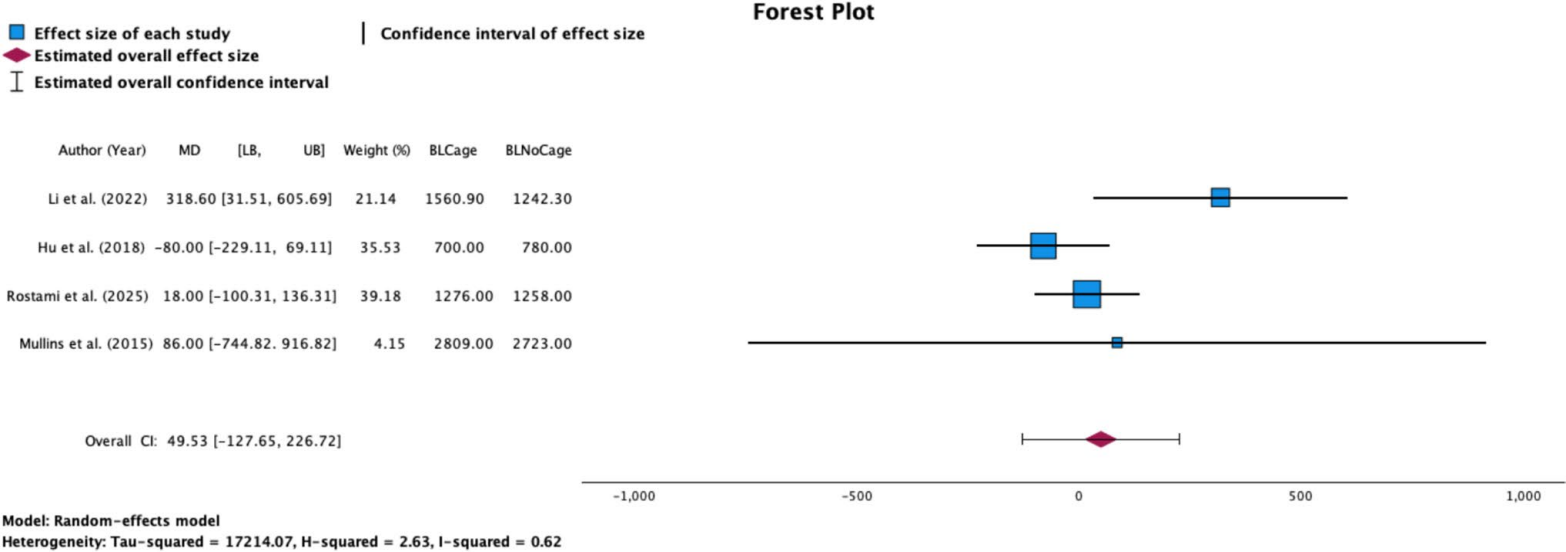
Random-effects model forest plot demonstrating the relationship between blood loss and cage use for four studies. Effect measures and CIs were calculated for both individual study outcomes and reported as overall pooled outcomes. *LB* lower bound, *UB* upper bound. Heterogeneity: Tau-squared = 17,214.07, H-squared = 2.63, I-squared = 0.62. The axis is shown using a linear scale

The FWM blood loss for osteotomies with cage use was 1,576.29 ± 483.58 ml compared to 1,513.64 ± 844.41 ml for cageless osteotomies. When examining intraoperative blood loss for high-grade osteotomies stratified by cage use via a random-effects meta-analysis, we found that there was no difference regardless of cage use (*p* = 0.584; Overall effect size: 49.53; 95% CI [− 127.65, 226.72]; Fig. 5**, **Table 4).

### Postoperative sagittal vertical axis and adequate correction by cage use

The FWM postoperative SVA for osteotomies with cage use was 5.20 ± 2.55 cm compared to 5.81 ± 2.50 cm for cageless osteotomies. When examining postoperative SVA for high grade osteotomies stratified by cage use via a random-effects meta-analysis, we found that osteotomies with cage use had significantly lower SVA compared to cageless osteotomies (*p* = 0.03; Overall effect size: − 0.90 cm; 95% CI [− 1.71 − 0.08]). Additionally, three studies reported whether final spinal alignment was adequate. Li et al. (2022) found that all high-grade osteotomies in both cage and cageless groups were deemed successful, with no significant differences between postoperative SVAs or Cobb angle at final follow-up (Table 2). Hu et al. (2018) found that both groups achieved similar spinal alignment following surgery [21]. Both groups saw significantly improved Cobb angles following surgery (Cage: 43.5 ± 14.8 to 7.8 ± 6.7, *p* < 0.01; no cage: 46.2 ± 16.7 to 8.8 ± 7.3), and final Cobb angles between groups were not significantly different. Finally, Mullin et al. (2025) found that ASD patients undergoing high grade osteotomies with cage use saw significantly improved fusion rates compared to patients who underwent cageless high grade osteotomies (Table 2) [23].

### Postoperative complications by cage use

We found that there was no significant difference in the incidence of rod fracture (6.94% vs 4.64%, *p* = 0.484; risk ratio (RR): 1.29; 95% CI [0.63, 2.66]), proximal junctional kyphosis (15.61% vs 14.9%, *p* = 0.753; RR: 1.08; 95% CI [0.68, 1.71]), or proximal junctional failure (4.05% vs 4.12%, *p* = 0.891; RR: 1.06; 95% CI [0.44, 2.58]) after high-grade osteotomies with cage use compared to cageless high-grade osteotomies, respectively. Additionally, we reported all-cause complication rates as events, since one patient may suffer multiple complications. We found that across 173 patients who received a cage, there were 98 events of complications. Across 194 patients without a cage, there were 124 events of complications.

### Postoperative patient reported outcomes by cage use

Three studies reported postoperative ODIs after high-grade osteotomy by cage use [22–24]. The FWM postoperative ODI for osteotomies with cage was 29.04 ± 5.84, and the FWM ODI for cageless osteotomies was 35.42 ± 5.46. When examining postoperative ODI for high-grade osteotomies stratified by cage use via a random-effects meta-analysis, we found that there was no difference regardless of cage use (*p* = 0.116; Overall effect size: − 5.22; 95% CI [− 11.74, 1.30]). Additionally, two studies reported postoperative VAS scores following high-grade osteotomy. Li et al. (2022) found that when treating kyphotic thoracolumbar osteoporotic fractures, there were no significant differences in postoperative VAS scores regardless of cage use (*p* > 0.05) (Table 4) [22]. However, Rostami et al. (2025) found that VAS scores were significantly better at 6 and 12 months postoperatively when using a cage in ASD patients who underwent high-grade osteotomies and spinopelvic fixation (*p* < 0.01) (Table 4) [24].

## Discussion

This systematic review and meta-analysis is, to our knowledge, the first to evaluate the impact of cage use in high-grade osteotomies for ASD correction, specifically focusing on its effect on sagittal alignment, patient-reported outcomes, and perioperative parameters. Our findings suggest that cage use in high-grade osteotomies modestly improves fusion rates and segmental lordosis compared to no cage, with a slight but statistically significant reduction in postoperative SVA by approximately 0.9 cm. While statistically significant, this difference is small and may fall within the range of expected intra-individual variation, suggesting that the observed improvement is unlikely to represent a clinically meaningful change. However, no major differences were found in regard to operative time, blood loss, or complication rates between the two groups, indicating that the addition of a cage does not substantially increase perioperative risk. While patient-reported outcomes, including the ODI and VAS scores, showed some trends favoring cage use, these differences were not consistent across studies and did not reach statistical significance. Ultimately, all patients showed meaningfully lower pain and disability scores following surgery regardless of cage use. These findings suggest that, although cage use offers modest improvements in alignment and fusion, its overall clinical benefit may be limited, particularly when considering the added complexity and potential cost. This analysis highlights the need for more robust prospective studies to better define the role of cages in high-grade osteotomies for ASD correction, especially in light of the minimal functional improvements observed in this cohort.

The clinical outcomes of high-grade spinal osteotomies for ASD, including PSO and BDBO, align closely with those achieved for other rigid spinal pathologies such as ankylosing spondylitis (AS) and congenital spinal deformities. In all cohorts, high-grade osteotomies provide substantial improvements in sagittal alignment and health-related quality of life, although these gains are tempered by high complication rates, including neurological and mechanical events [4, 6, 13, 25]. Specifically, ASD patients often face a higher risk of pseudoarthrosis and mechanical complications, partly due to older age, higher BMI, and comorbidities. Conversely, fusion rates in AS are typically near-universal, owing to the ankylosed nature of the spine, and in congenital deformities, fusion rates remain robust in younger populations, though nonunion and hardware failure risks rise with more severe deformities and higher osteotomy grades [4, 5, 13, 25]. Alignment correction in ASD is comparable to AS, with substantial improvements in SVA, but up to 27% of patients may experience loss of correction at two years, especially those with higher BMI or suboptimal implant choices [26, 27]. AS shows dramatic and durable correction, typically exceeding 30° per osteotomy, with minimal loss over time. In congenital cases, large corrections (~ 53%) are similarly stable, although complication rates are heightened with increasing osteotomy severity [26, 27]. These findings suggest that while ASD treatment outcomes are broadly comparable to those for AS and congenital deformities, ASD patients face unique challenges—particularly regarding aging and comorbidities—that necessitate careful preoperative planning, optimal fusion adjuncts (e.g., interbody cages, pelvic fixation, or biologics), and meticulous postoperative management to maximize correction and minimize complications.

Importantly, the complication profiles between the cage and no-cage groups in our analysis were comparable, with both groups exhibiting similar rates of rod failure, proximal junction kyphosis, and proximal junction failure after surgery. These observed trends suggest that the addition of a cage does not dramatically worsen the perioperative risk, though the overall rates of complications remain relatively high in both groups, reflecting the inherent complexity of high-grade osteotomies. An important consideration to bear is that based on the available data, it appears that the addition of cages may not meaningfully hamper the safety profile of the high-grade osteotomy while still offering comparable correction that is important.

The fiscal implications of cage use within this procedure are an important factor to consider given the similar clinical picture regardless of cage use in the current study. Adding an interbody cage to high-grade spinal osteotomies, such as Schwab grade 3 and 4 procedures, increases the direct procedural costs, primarily due to the price of the cage and the additional instrumentation required. The cost of a single interbody cage can range from several thousand to over 10,000 US dollars ($), depending on the material and manufacturer, contributing to the already substantial costs of complex spinal deformity surgeries, which in the United States can exceed $70,000–$150,000 for the index hospitalization [28–30]. Furthermore, cage use tends to increase operative time according to the literature, necessitating additional resources such as fluoroscopy and specialized instruments, which further elevate both direct and indirect costs. Despite the increased upfront cost, higher-cost surgeries—including those with interbody cages—have been seen to be associated with superior patient-reported outcomes at 2 years in the literature, suggesting that the additional expenditure may be justified in selected patients with severe deformity or high risk of nonunion [23]. Cost-effectiveness analyses indicate that while the initial expense is higher, the incremental cost per quality-adjusted life year is not significantly different from other complex fusion strategies, and the overall value may be improved if revision rates are reduced [28, 31].

Our findings suggest that the addition of a cage yields modest improvements in clinical outcomes, such as a significant but small reduction in SVA and higher fusion rate trends. Thus, it is prudent to question if the improvement in alignment (approximately 0.9 cm in SVA) and fusion rates, while statistically significant, justify the added expense in all patients. The duration and complication profile of this procedure with a cage do not appear to be meaningfully different, suggesting that the clinical benefit must be weighed against the financial and clinical considerations. Therefore, while the initial expense of adding a cage to high-grade osteotomies is considerable, its cost-effectiveness may be more favorable in specific patient populations at high risk for nonunion or mechanical failure, where long-term benefits such as reduced revision rates could potentially justify the additional upfront cost.

This study has several notable strengths. It represents the first pooled meta-analysis of four studies comparing the use of interbody cages in high-grade spinal osteotomies for ASD. This provides a unique contribution to the literature, offering a clearer understanding of the perioperative characteristics and clinical outcomes associated with cage use in this complex surgical population. Additionally, all included studies were comparative in nature, with moderate quality ratings, which allows for a more robust analysis. The inclusion of key perioperative parameters, such as operative time, blood loss, and complication rates, adds significant clinical value to our findings. However, there are several important limitations. The retrospective design of the included studies introduces potential biases, such as selection bias and confounding factors, which could influence the results. Despite the inclusion of moderate-quality studies, the overall evidence remains non-randomized, and the small sample sizes limit the statistical power, especially given that most outcomes had moderately low event rates. Furthermore, while the complication rates were similar between the cage and no-cage groups, the inability to perform a formal meta-analysis of these complications means our conclusions are based on observed trends, which may not fully capture the true risk of adverse events. The heterogeneity across studies in terms of patient populations, osteotomy types, and follow-up duration also limits the generalizability of the results and contributes to the variance within the meta-analysis model. Finally, because all included studies were retrospective and non-randomized, unmeasured confounders such as surgeon intent, patient-specific deformity characteristics, and perioperative management may have influenced the observed associations between cage use and outcomes. Despite these limitations, this study provides the first comprehensive meta-analysis on this topic, offering valuable insights into the comparative effectiveness of cage use in high-grade osteotomies for ASD.

## Conclusion

In this systematic review and meta-analysis, we present the first comprehensive pooled analysis of interbody cage use in high-grade osteotomies for ASD. Our findings suggest that cage use may be *associated* with modest improvements in sagittal alignment and fusion rates, as evidenced by a small reduction in postoperative SVA and a trend toward higher fusion rates. However, the magnitude of SVA reduction was minimal and likely not clinically significant, underscoring that these results should be interpreted as limited statistical associations rather than definitive clinical advantages. Furthermore, given the observational nature of the included studies, these associations should not be interpreted as evidence of causation, and surgeon selection, patient factors, and institutional variability may all contribute to these outcomes. No significant differences were observed in operative time, blood loss, or patient-reported outcomes. Both groups showed substantial improvements in ODI, with no clear advantage for cage use in terms of functional outcomes. The complication profile of using cages was similar and within acceptable margin when compared to the cageless approach. While these findings suggest that cage use may offer some benefit in terms of alignment and fusion, the clinical significance of these improvements remains uncertain. Patients at high risk for nonunion or mechanical failure may be of greater benefit from the addition of cages. Further prospective studies with larger, more homogeneous cohorts are necessary to better define the role of interbody cages in high-grade ASD correction. Future research should aim to clarify whether the observed modest benefits translate into meaningful long-term outcomes for patients, including the potential for reduced revision rates and improved overall spine function.
